# Eight-Channel Multispectral Image Database for Saliency Prediction

**DOI:** 10.3390/s21030970

**Published:** 2021-02-01

**Authors:** Miguel Ángel Martínez-Domingo, Juan Luis Nieves, Eva M. Valero

**Affiliations:** Department of Optics, University of Granada, 18071 Granada, Spain; martinezm@ugr.es (M.Á.M.-D.); jnieves@ugr.es (J.L.N.)

**Keywords:** attention, multispectral database, saliency, eyetracker, computational vision, spectral images, color images

## Abstract

Saliency prediction is a very important and challenging task within the computer vision community. Many models exist that try to predict the salient regions on a scene from its RGB image values. Several new models are developed, and spectral imaging techniques may potentially overcome the limitations found when using RGB images. However, the experimental study of such models based on spectral images is difficult because of the lack of available data to work with. This article presents the first eight-channel multispectral image database of outdoor urban scenes together with their gaze data recorded using an eyetracker over several observers performing different visualization tasks. Besides, the information from this database is used to study whether the complexity of the images has an impact on the saliency maps retrieved from the observers. Results show that more complex images do not correlate with higher differences in the saliency maps obtained.

## 1. Introduction

Human observers are able to process a considerable number of visual stimuli. However, they focus their attention on certain areas of the scene or some objects that are either markedly distinct from their surroundings (e.g., mature orange fruits that hang from an orange tree) or relevant for the task they are performing (e.g., traffic lights when the subject is planning to cross a street). In the first example, the subject’s visual attention mechanisms are driven by the inherent features of the stimuli (differences in shape and color from the surroundings). This is a typical instance of bottom–up visual attention [1]. In the second example, the subject’s task determines primarily which stimuli are relevant or not (which is usually described in the framework of top–down visual attention mechanisms [2]).

An entire branch of computational vision has originated from the interest in characterizing and mainly also predicting the areas of interest for a given subject when looking at a scene (i.e., salient areas). Moreover, numerous models have been developed and tested for their performance in two main tasks: saliency detection (when the model’s aim is to predict and segment the most salient object in the scene [3,4,5,6]) and saliency prediction (when the model’s aim is to predict the areas to where the subject’s gaze will be directed for longer periods of time [5,7,8,9]).

In general, saliency prediction is considered to be a harder task than saliency detection. Both kinds of visual attention models (saliency detection and saliency prediction) have had similar trends in their evolution. The initial period was marked by the extended use of visual features of different types (edges, orientation, color [10]) previously selected and computed from the original image, and transformed with different image processing operations (some of them inspired by the way visual stimuli are processed by the brain) until they yielded the model’s outcome. More recently, the trend in visual attention models, as in many other areas of computer vision, has been to use convolutional neural nets (CNNs [8,9]) to automatically find the best features and operations to be performed to get the highest accuracy in the model’s outcome and even develop specific models for saliency prediction in video scenes [11].

The accuracy of the models is determined by using a database of annotated images (with manual segmentation for the saliency detection task) or a database that comprises as well the gaze fixation maps or heat maps for the given image set, obtained usually with the aid of a fixation tracking device or eyetracker [12] for the saliency prediction task. Besides the database, saliency detection or prediction accuracy requires the use of specific metrics, some of them specifically designed for the task. Some widely used metrics for this purpose are the normalized scanpath saliency (NSS [13]), the area under the curve (AUC) in its different implementations [14,15,16,17], and the related F-factor [18].

For color (RGB) images, there are numerous kinds of saliency image databases, including the MIT Saliency Benchmark [19], SALICON [20], ECSSD [21], THUR15K [22], DUT-OMRON [23], SED [24], and HKU-IS [25], to name a few of the most popular ones. The number of images ranks from a few hundred to more than 10,000. Some are specific for saliency detection and/or prediction. With CNN-based models, it is quite important to ensure that enough images are used for an adequate training, and that they are sufficiently representative of all conditions in which the model is intended to be used, to avoid the tendency to overfit.

Recently, saliency models have been expanded to accept multispectral images as input [26] (like RGB + Near Infrared (NIR) [27] or RGB + thermal [28]), or even hyperspectral images [15,29]. In their recent study, İmamoğlu et al. [29] proposed a CNN-based model that generates its own ground truth for hyperspectral images. This is done due in part to the scarcity of spectral databases annotated specifically for saliency detection, which they mention in the introduction. However, to our knowledge, still today there is not any publicly available database with multispectral (more than four channels) or hyperspectral images and their corresponding heat maps, which could be used for visual saliency detection or prediction model development and testing.

This study aims to cover this gap by presenting a database of multispectral images of urban scenes (with eight spectral channels, six in the visible range and two in the NIR range) and their corresponding saliency maps. The database is composed of two sets of images. The first set contains 1147 multispectral images together with their RGB versions, as well as the raw heat maps of 10 observers recorded with an eyetracker while performing free viewing. The second set contains an additional set of 136 higher-resolution multispectral images together with their RGB versions, as well as two sets of heat maps: one generated during free viewing and the other during category counting. For both sets, the raw heat maps and the filtered heat maps are provided observer-wise and accumulated (see Section 2.2 for explanation). In this study, we do not aim to develop a specific model for saliency prediction with multispectral images. The database provided can be used for that purpose, and we expect it will be in future studies.

Besides presenting the database, which, to our knowledge, is the first and most comprehensive up to date for multispectral saliency prediction testing, this study will thoroughly analyze the differences between free-viewing and specific-task (category counting) saliency maps and introduce a correlation study between saliency map comparison metrics and image complexity metrics. Besides, an example comparison is also performed with an existing saliency prediction model [30] using both RGB images and the different spectral bands of the multispectral images.

The paper is structured as follows: in Section 2, we present the methods used and the experiments performed to build the database; in Section 3, we present the results of the experiments performed with the database; and finally, we describe the most relevant conclusions reached in Section 4.

## 2. Methods

### 2.1. Multispectral Image Capturing

**Figure 1:**
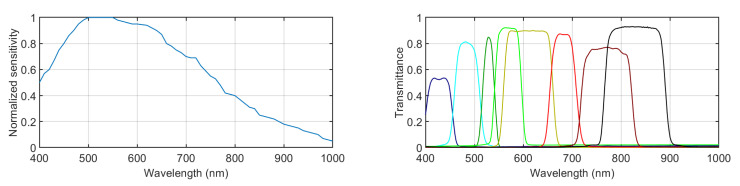
(**Left**) Normalized spectral sensitivity of the sensor. (**Right**) Spectral transmittance of the 8 selected filters.

The multispectral images were captured using a filter-wheel-based multispectral camera model, PixelTeq Spectrocam VISNIR (PixelTeq Ltd., Largo, FL, USA) [31]. This camera features a silicon sensor that is sensitive from roughly 400 to 1000 nm (its normalized spectral sensitivity is shown in Figure 1, left). The image full size is 2456 × 2058 pixels. The objective is a Zeiss model, Milvus 2.8/15 (Zeiss GmbH, Oberkochen, Germany) [32], with a variable focal length ranging from 2.8 to 15 mm and manual focus. The aperture used was 5.6 for all the captures. The exposure times were manually selected individually for each capture and filter. They were adjusted by monitoring the histogram of the whole image, setting the maximum possible exposure time yet avoiding any saturation. This camera is synchronized with a filter wheel with eight slots. Eight band-pass filters were selected, covering the visible and near-infrared region of the spectrum. The spectral transmittances of these selected filters are plotted in Figure 1, right. Table 1 shows the central wavelength and the bandwidth of each filter.

In each capturing sequence, three images were captured and averaged for each filter in order to reduce the impact of noise. Exposure times ranged from 1 ms to 2 s, depending on the scene and the filter.

Each eight-band multispectral image had a bit depth of 12 bits. It was stored in a floating point format with values ranging from 0 to 1. Besides, three channels were selected to become red, green, and blue (R, G, and B) channels of the color images. They were the ones corresponding to filter numbers 6 (red), 4 (green), and 2 (blue). Each color channel was individually normalized to range (0, 1) as a manual white balance. Alterations in the apparent color of the objects introduced by the selection of these three particular channels as RGB bands did not compromise the tasks performed in the experiments presented in this study. The final appearance of the RGB images built was pretty natural, and no color artifacts were introduced due to the selection of these spectral bands as red, green, and blue channels, as can be seen in the example RGB images presented in Section 2.4.

The color image rendered was stored in an 8-bit jpeg format with no compression.

### 2.2. Gaze Data Recording

A total of 104 scenes were captured using the method explained in Section 2.1 in two capturing campaigns. In the first one, 44 scenes were captured, and in the second round, 60 extra scenes. After dividing the resulting color images as explained in Section 2.4 (which resulted in 136 and 1137 images, respectively), they were displayed in a calibrated monitor model, Eizo Color Edge CG277 (Eizo Nanao Corp., Hakusan, Japan) [33]. The details of the experiments, such as display time and size, are explained in detail in Section 2.4. The spectral distribution of the object in the real scene cannot be reproduced by the monitor display, but this is not a relevant limitation for the set of experiments performed since saliency is not necessarily determined by the spectral distribution of the object, but it can be conditioned by the perceived color. The color fidelity of the monitor was ensured by the calibration performed. The binocular gaze data were recorded using an eyetracker model, Tobii Pro X2 60 compact (Tobii Technology, Danderyd, Sweden) [12], with a binocular gaze sampling frequency of 60 Hz, an accuracy of 0.4o, and a precision of 0.34o according to the manufacturer. The eyetracker was set at the bottom edge of the monitor, and the distance from the monitor to the observers was 60 cm. A total of 14 different observers participated in the two experiments explained in Section 2.4. Six observers were females, and 8 were males. Ten observers were in the age range from 20 to 40 years and 4 from 40 to 60 years. Besides, 10 of the observers were naïve. The study was conducted in accordance with the Declaration of Helsinki, and the protocol was approved by the Ethics Committee of the University of Granada (1741/CEIH/2020).

### 2.3. Building of the Fixation Maps

The eyetracker device used allowed the recording of a huge amount of information during the gaze experiments. Separately for each eye, the device sampled its position 60 times per second. In each of these samples, the device recorded not only the horizontal and vertical coordinates of the gaze point but also the type of movement the eye was doing (fixation or saccades), the time stamp, the pupil size, the distance to the screen, and the validity of the record. All this information ended up in a big amount of data to be processed before retrieving the final fixation map. A heat map is a three-dimensional array. The first two dimensions correspond to the horizontal and vertical coordinates of the image displayed in the monitor (X and Y, respectively). The third dimension is related to the fixation time in those spatial coordinates. This fixation time is at the same time related to saliency. The more salient the content of a given area of the image is, the longer the fixation time within the spatial coordinates of this region of interest is.

**Figure 2:**
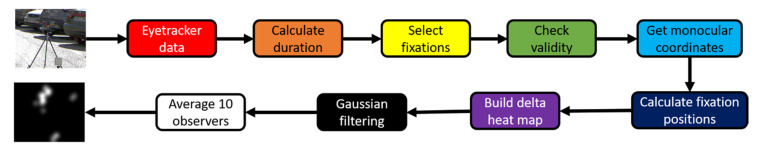
Flowchart followed for building heat maps from eyetracker recorded data.

Out of the available information, the heat maps were built using the data from the fixations only. Figure 2 shows a flowchart with the steps followed to get the final heat maps. From the eyetracker data for a given scene, the first step was to calculate the duration of the eye movements recorded. Then, we selected the movements that qualified as fixations (not saccades). We then checked the validity of the movements and excluded those that were nonvalid, as explained below. The following steps are the computation of the fixation positions and the building of the heat maps with delta functions in the fixation locations. Finally, Gaussian filters were added to this preliminary heat map, and the average heat map of the 10 observers was calculated.

The duration of each eye movement was calculated as the difference between the time stamps of two consecutive movements. Then every movement that was not a fixation was filtered out. After that, also those fixations whose binocular validities were not positive were filtered out. Validity is a binary variable whose value is 1 (positive) if both eyes are sufficiently opened and the eyetracker is detecting them correctly. Otherwise, its value is 0 (negative).

**Figure 3:**
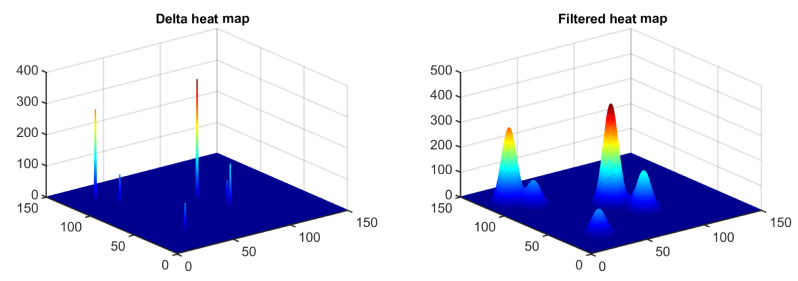
Sample heat map with a size of 150 × 150 pixels. (**Left**) Raw delta heat map. (**Right**) Same heat map after applying the Gaussian filtering.

With the remaining valid fixations, the monocular spatial coordinates X and Y were retrieved. Since these coordinates were not the same for both eyes, the mean position was calculated, and this was used as the real fixation position. With these spatial locations and their corresponding fixation times, we built a raw heat map placing deltas (step 3D functions of 1 × 1 pixel width) whose heights equaled the fixation time in those positions. Once these preliminary heat maps were built, we convolved them with a two-dimensional Gaussian filter of radius R = 100 pixels and standard deviation σ = 20 pixels. Thus, the full width at half maximum (FWHM) equaled 48 pixels. Since the spatial resolution of the monitor was 4.26 pixels/mm, the angle subtended by the Gaussian curve at half maximum was 1.075o at the viewing distance used in the gaze recording experiments. This was in accordance with previous works by other authors [34]. In Figure 3, a surface plot of a sample heat map with a size of 150 × 150 pixels is shown before (left) and after (right) applying the Gaussian filtering.

**Figure 4:**
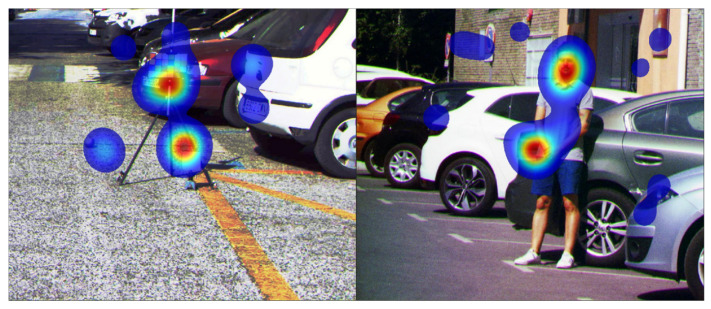
Two examples of accumulated heat maps overlaid on top of their corresponding images.

For each observer, the raw delta heat maps and the filtered heat maps were provided, and for each image, the accumulated heat map was calculated, summing up all deltas from all observers. The raw and filtered versions of these accumulated heat maps were provided as well. Both the raw delta heat maps and the final filtered heat maps were provided in case the users would like to create a differently filtered heat map. Figure 4 shows two examples of accumulated filtered heat maps overlaid on top of their corresponding RGB images.

### 2.4. Experiments

As explained in Section 2.1, two different capturing campaigns were carried out. Therefore, two different experiments were performed with the two different sets of images retrieved from those campaigns. Their details are explained in Section 2.4.1 and Section 2.4.2. Moreover, a third experiment was performed comparing the performance of an existing saliency prediction model using RGB images vs. using spectral band images (see Section 2.4.3).

#### 2.4.1. Small Image Set: Free Viewing + Categorization

Using the 44 scenes captured in the first campaign, a double experiment was performed to assess whether the saliency information provided by the heat maps was different depending on the task that was asked of the observers. Each image was divided into 4 sub-images (1228 × 1029 pixels each), and then those whose content was not significant (e.g., the whole sub-image represents only an empty blue sky) were deleted, resulting in a set of 136 sub-images. The display size on screen was 290 × 240 mm. Each image was displayed for 6 s, and between images, a central white cross was displayed for 2 s. The observers were asked to fix their gaze at the center of this cross in order to have an initial central gaze point for every image.

**Figure 5:**
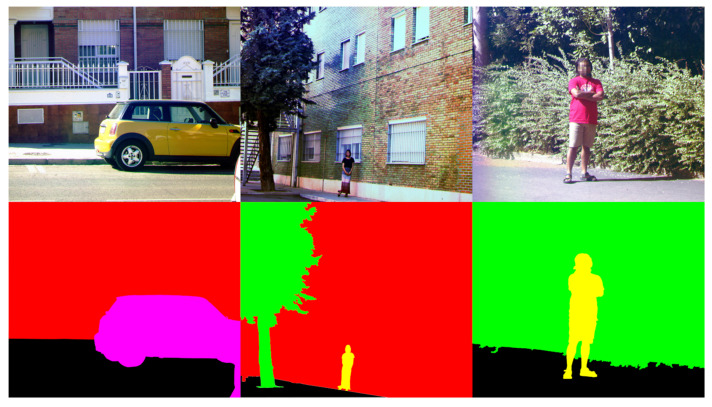
Top row: 3 example RGB images used in the experiment. Bottom row: manual segmentation of the top-row images. In the bottom left, 2 categories are present, which include building (red) and vehicle (pink). In the bottom center, 3 categories are present: vegetation (green), building (red), and people (yellow). In the bottom right, 2 categories are present: vegetation (green) and people (yellow). In all cases, ground is represented in black, and no category is assigned to it.

In the first round of this experiment, the observers were asked to just freely look at the images with no purpose other than to see what was the content in them (free-viewing task). In the second round, the observers were asked to count the number of categories present in the scenes (categorization task). There were seven categories: buildings (including every object attached to a building, such as pipes, antennas, etc.), people, vehicles, vegetation, traffic signs (both vertical ones and those painted on the ground), urban furniture (such as bins, garbage containers, streetlights, benches, fences, sewers, etc.), and other (whatever was not included in the previous categories). The observers were asked to disregard both the asphalt or ground and the sky when counting the categories. In Figure 5, three example RGB images are shown with a manually made segmentation (for illustration purposes only), indicating the different categories present on them. The subjects were asked to voice the number of categories found in each image after it was displayed.

#### 2.4.2. Large Image Set: Free Viewing

Using the 104 scenes captured in the two campaigns, a second simple experiment was performed. Now the images were divided into 16 sub-images (614 × 514 pixels each), and those whose content was not significant were deleted, resulting in a set of 1147 sub-images. The display size on screen was 145 × 120 mm. Each image was displayed for 4 s, and between images, a central white cross was displayed for 1.5 s for the same purpose as in the first experiment.

The whole experiment took 38 min and 8 s per observer (total image display time). Ten observers performed the experiment, and for each image, the 10 observer-wise heat maps and the accumulated heat maps were stored.

Now the observers were only asked to freely look at the images to see the content in them. For each observer, a total image display time of 1 h, 45 min, and 9 s was taken. The observers could complete the whole experiment in different sessions of variable lengths since they could control the ending of each session. Each image was displayed only once for each observer.

#### 2.4.3. Saliency Prediction Comparison: RGB vs. Spectral

Using the 136 RGB and multispectral images created for the experiment described in Section 2.4.1, a saliency prediction model was used in both the RGB images and the different spectral channels of the multispectral images separately. The saliency prediction model was RARE [30]. Different metrics (as explained in Section 2.5) were used to study the statistics in the differences between using RGB images and single-band spectral images as input for the saliency prediction model.

### 2.5. Heat Map Comparison Metrics

A correlation study was carried out between heat map comparison metrics and image complexity metrics to test whether the hypothesis that more complex images increase differences in the way observers look at the images is true. Additionally, inter-experiment and inter-observer (intra-experiment) comparisons were made among heat maps. These metrics are designed to compare a heat map with a ground-truth saliency map. In our case, heat maps were compared two by two. All the metrics were computed using one of the compared heat maps as ground truth and the other as test heat map. The heat map comparison metrics studied [26] are the following:

- Area under the curve (AUC): three different versions of this metric were computed: AUC-Borji (AUCB) [16], AUC-Judd (AUCJ) [14], and shuffled AUC (sAUC) [16]. For different values of threshold in the heat map, true positives and false positives are computed by using the other heat map.
- Normalized scanpath saliency (NSS) [13]: averaged normalized saliency at the ground-truth location. This solves the issue existing in AUC methods of not penalizing low-valued false positives.
- Information gain (IG) [35]: compares two heat maps, taking into account the similarity of the probability distribution function and the heat map acting as ground truth.

Although there are many different kinds of metrics developed or used for heat map comparison [36], we chose this particular set of metrics for the following reasons: their use is quite common in the literature, the ideas underlying their design are different, and so they allow us to compare different aspects of the heat map intensity distribution.

### 2.6. Image Complexity Metrics

Complexity metrics have been used in early studies to analyze the representation of scene properties directly related to the subjective impression—aesthetic—of images [35]. In order to try to correlate the inter-observer and inter-experiment differences found on the heat maps with image complexity, the following five different image complexity metrics [37] were calculated from the RGB images shown during the viewing experiments:

- Self-similarity: self-similarity compares the histogram of gradients (HOG) across different equally sized sub-images of the original image [37]. The HOG feature is calculated for each sub-image in the pyramid using the histogram intersection kernel [38]. By comparing the HOG features of each sub-image at level 3 with those of the entire image at level 0, the self-similarity of an image is calculated as shown in Equation (1).
  (1) $$M_{SeSf}\left(I,L\right)\;=\;median(HIK\left(h\left(S\right),\;\left(h\left(Pr\left(S\right)\right)\right)\right)|\;P_{r}\left(S\right)\;\in \;Sections\left(I,L\right))\;$$
  where *I* represents the image, *L* represents the three pyramid levels used, *h*(*S*) is the HOG value for a sub-image, and *P_r_(S*) corresponds to the parent of sub-image S [39].
- Complexity: by computing first the maximum gradient magnitudes in the image channels, the gradient image *G_max_* is generated as shown in Equation (2).
  (2) $$G_{max}\left(x,y\right)=max\left(\left\|\nabla I_{R}\left(x,y\right)\|,\|\nabla I_{G}\left(x,y\right)\|,\|\nabla I_{B}\left(x,y\right)\right\|\right)$$
  where $\nabla I_{R}\left(x,y\right)$, $\nabla I_{G}\left(x,y\right)$, and $\nabla I_{B}\left(x,y\right)$ are the gradients at pixel (*x*, *y*) for each *R*, *G*, and *B* image component. Finally, the complexity of an image is computed as the mean norm of the gradient across all orientations over G_max_(*x*, *y*) [37], as shown in Equation (3).
  (3) $$M_{Co}\;\cdot \underset{\left(x,y\right)}{\sum }G_{max}\left(x,y\right)$$
- Birkhoff-like metric: this metric computes the amount of effort the human visual system has to put into the processing of an image. Following on from [37], the Birkhoff metric is defined as the ratio between self-similarity and the complexity metrics previously explained (see Equation (4)).
  (4) $$M_{BLM}=\frac{M_{SeSf}}{M_{Co}}$$
- Anisotropy: calculated as the variance of all the HOG values at level 3 as explained in [37]. This metric gives an idea about how the Fourier spectrum is more or less uniform across orientations (that is, less anisotropic).
- Entropy: this is a statistical measure of the randomness present in an image. The higher is its value, the more complex the image is supposed to be [39,40].

## 3. Results

First, we analyzed the similarity between the fixation mapsin the case of inter-observer and inter-experiment comparisons. Next, the results of the correlation of the heat map comparison metrics with the image complexity metrics would be shown.

### 3.1. Inter-Observer and Inter-Experiment Heat Map Comparisons

**Figure 6:**
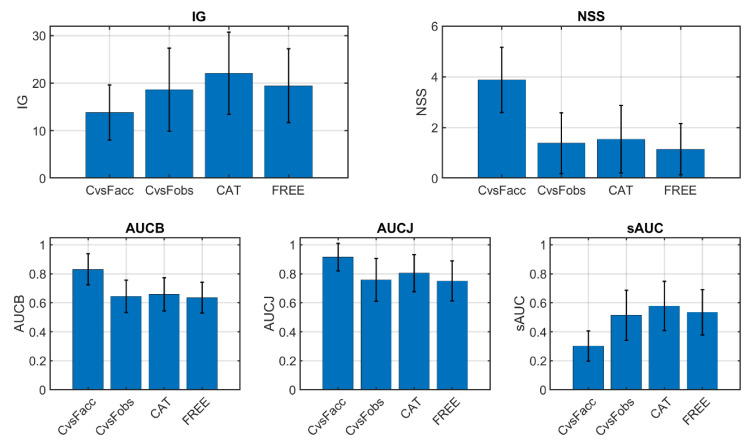
Inter-experiment and inter-observer heat map comparison metrics. CAT: category counting experiment. FREE: free-viewing experiment. CvsFAcc: inter-experiment comparison with accumulated heat maps. CvsFobs: inter-experiment comparison with individual observer data.

The five heat map comparison metrics explained in Section 2.5 were computed to compare the heat maps obtained in the small image set experiments explained in Section 2.4. The inter-observer (comparing different observers performing the same task) and inter-experiment (comparing the same observer performing different tasks) comparisons were made, as explained in the following. Graphical results are shown in Figure 6. Since all metrics indicate heat map similitude, the higher their values are, the more similar the heat maps are.

Inter-observer comparison: heat maps were compared between different observers performing the same task two by two. Since there were 10 observers, there existed 45 different two-by-two comparisons. All of them were made, and the average metric values are shown in Figure 6. Since there were two tasks (categorization and free viewing), their results are plotted separately (CAT and FREE, respectively).

Inter-experiment comparison: here, the heat maps were compared in two different ways. First of all, for each image, all gaze data from all observers were accumulated in a single raw delta heat map, and then filtered accumulated heat maps from the categorization and free-viewing experiments were compared (C vs. Facc in Figure 6). On the other hand, for each image, the filtered heat maps of one observer were only compared with itself between the two experiments. Therefore, for each image, we got 10 comparison metric values (corresponding to the 10 observers), and we plot their average in Figure 6 (C vs. Fobs).

As can be seen in Figure 6, inter-observer heat map comparison metrics were very similar in both experiments (CAT and FREE), although slightly higher for CAT. For the inter-experiment cases, the accumulated comparison (C vs. Facc) yielded more different results compared with the inter-observer values, being higher for NSS, AUCB, and AUCJ and lower for IG and sAUC. The metric values for the C vs. Fobs comparison were similar to those for the CAT and FREE comparison, but the C vs. Facc comparison yielded either higher or lower results. This can be explained if we consider that in the C vs. Facc comparison, the accumulated heat maps tended to be fuller (they had less empty or black pixels) than those in the other comparison conditions. Then, it was harder to gain more information by comparing CAT and FREE experiments. However, at the same time, metrics that relied mostly on gaze positions (like NSS) would tend to have higher values for fuller heat maps. The nonshuffled versions of the AUC metric (AUCB and AUCJ) presented the same effect as NSS, which was corrected in the shuffled version. The lower value for sAUC might be due to the relatively more abundant fixations in the center region of the accumulated heat maps. The sAUC metric tended to give more weight to peripheral fixations than to central fixations.

### 3.2. Correlation with Image Complexity Metrics

**Figure 7:**
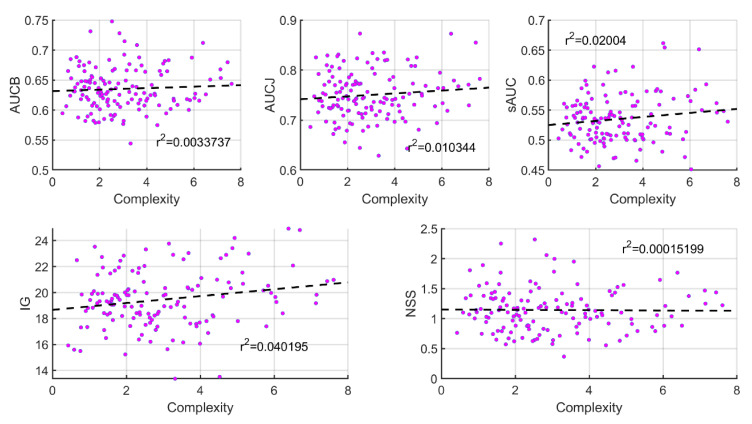
Correlation plots comparing heat map comparison metrics with the Complexity metric for the inter-observer free-viewing case. Linear fits are shown as dashed lines, and the r^2^ correlation coefficients are shown as insets.

The correlation between the five heat map comparison metrics explained in Section 2.5 and the five image complexity metrics explained in Section 2.6. was calculated. This was done for both inter-observer and inter-experiment cases. In all cases, a linear fit was computed, as well as the r^2^ correlation coefficient. Figure 7 shows an example of where the five heat map comparison metrics are plotted against Complexity for the free-viewing inter-observer case. As can be seen, r^2^ coefficients are very low, which indicates no correlation between metrics. The same trends were found for the rest of the comparisons, the highest r^2^ correlation coefficient being found to be 0.046 between sAUC and Anisotropy in the free-viewing inter-observer case and between NSS and anisotropy for the observer-wise inter-experiment case.

Table 2 shows the mean r^2^ values (and standard deviation) for each heat map comparison metric across all image complexity metrics.

As shown in Table 2, r^2^ correlation coefficients are very low for all experiments and all metrics studied. These results refute the hypothesis that more complex images result in higher differences in the way observers look at the images.

### 3.3. Example of Saliency Prediction Comparing RGB and Single-Band Spectral Images

**Figure 8:**
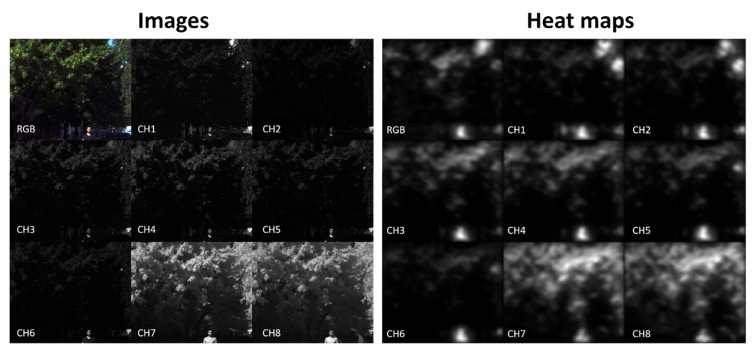
(**Left**) Example images fed to the RARE saliency prediction model. (**Right**) Heat maps generated for those images. RGB stands for the color image, and CHX for channel X of the multispectral image.

**Figure 9:**
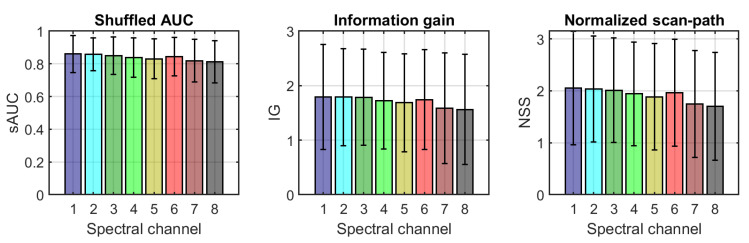
From left to right: mean (bars) and standard deviation (error lines) for sAUC, IG, and NSS metrics over the 136 images comparing the heat maps generated by the RARE model from the RGB images and each of the 8 spectral channels of the multispectral images. The colors of the bars match the false colors of the transmittance plots for each channel in Figure 1, right.

As explained in Section 2.4.3, the 136 images of the small images’ dataset were used as input for the RARE saliency prediction model [30]. On the one hand, the RGB images were fed to the model, and their heat maps were calculated. On the other hand, the eight channels of the multispectral images were separately fed to the same model, and a heat map was obtained for each scene and spectral band. Three metrics were computed comparing the heat maps of each of the spectral bands with those of the corresponding RGB images. The 9 images (RGB and eight channels represented in grayscale) and the nine heat maps of an example scene are shown in Figure 8. The mean value and the standard deviation of the three metrics used are shown in Figure 9.

There are some clear differences in the information contained in the images corresponding to each spectral band with respect to the RGB image (see, for example, in Figure 8, left, how the small patch of sky in the upper-right corner is clearly visible in the first two bands, but it is not visible in bands 3–6; instead, the vegetation is clearly visible in the images corresponding to bands 7 and 8 due to the well-known spectral reflectance rise in the NIR region for plants due to their chlorophyll content). These differences are reflected also in the heat maps extracted by RARE, as shown in Figure 8 (right).

The results of the comparison between RGB and single-band heat maps are shown in Figure 9 for the three metrics tested. The mean values for the 136 images range from 0.81 to 0.86 for sAUC, from 1.59 to 1.76 for IG, and from 1.70 to 2.05 for NSS, showing that there is a certain degree of similarity between the maps, but they are still far from being nearly identical. The IG results are computed as the difference between the amount of information in the RGB image and the amount of information in the single-band images, and the fact that they are positive reflects that the heat map derived from the RGB image contains a higher amount of information. The trend for all the three metrics is to show slightly less similarity between the RGB heat map and the single-band heat maps for bands 3 to 5 and for the last two bands in the NIR, which is as expected given the difference found in the intensity distributions that are fed to the RARE model. We performed a one-way ANOVA to see whether these differences were statistically significant. The results of this analysis show that the differences found are significant for sAUC (F = 2.83, *p* = 0.0063) and NSS (F = 2.21, *p* = 0.0312) but not significant for the IG metric (F = 1.29, *p* = 0.253).

## 4. Discussion and Conclusions

This article presents a database of eight-channel visible and near infrared (VIS+NIR) multispectral images of urban outdoor scenes together with saliency maps measured using an eyetracker device on different observers. The database can be downloaded and is made publicly available from [41]. Two sets of images are included: one with free-viewing saliency maps only and another one with task-driven saliency maps as well. This database is, to our knowledge, the first one freely available about multispectral images with gaze data in order to adapt existing saliency models from RGB images into spectral images or develop new ones on the basis of visible and near-infrared multispectral images. The RGB version of the multispectral images are included in the database as well.

Besides, three example experiments were presented as instances of what this database can also be used for. Results from these experiments show that the differences found in the saliency maps were not significantly higher or lower if we compare saliency maps between different observers or between different tasks performed by the same observer. On the other hand, we also found that the complexity of the images was not related to inter-observer variability, meaning that more complex images do not yield higher differences in the way observers look at them. This lack of correlation between image complexity metrics and saliency map comparison metrics happened in both inter-observer (different observers performing the same task) and intra-observer (same observer performing different tasks) comparisons. We also performed a comparative analysis of the heat maps obtained with the RARE model for the RGB image and the different spectral bands. Our previous study [26] showed that using the information contained in the multispectral images and adapting the saliency models, there was an improvement in the model performance. The new analysis presented in this study used only individual bands and showed that each band was not enough to adequately predict the saliency maps obtained with the RGB image. Our interpretation of the results of the two studies is that it is crucial to design effective ways to combine and extract the extra information contained in the multispectral image to be able to exploit it adequately for saliency prediction. In other words, we found that, as expected, one band is not enough to reach results comparable to an RGB image, but the combination of several bands in different ways can add to the model prediction capability.

## Figures and Tables

**Table 1:** Central wavelengths and bandwidths of the selected filters.

Filter #	1	2	3	4	5	6	7	8
**λ (nm)**	425	482	530	570	615	680	770	833
**BW (nm)**	50	56	24	50	100	50	102	125

**Table 2:** Mean r^2^ correlation coefficients (and standard deviation) for each heat map comparison metric across all image complexity metrics.

Metric	AUCB	AUCJ	sAUC	NSS	IG
C vs. Fobs	0.0214 (0.0141)	0.0170 (0.0160)	0.0193 (0.0138)	0.0349 (0.0208)	0.0048 (0.0047)
C vs. Facc	0.0074 (0.0074)	0.0021 (0.0020)	0.0062 (0.0080)	0.0066 (0.0081)	0.0076 (0.0114)
CAT	0.0041 (0.0045)	0.0062 (0.0096)	0.0012 (0.0019)	0.0032 (0.0054)	0.0009 (0.0013)
FREE	0.0061 (0.0096)	0.0053 (0.0062)	0.0086 (0.0102)	0.0089 (0.0167)	0.0119 (0.0137)

## Data Availability

The complete database is available for free download at http://colorimaginglab.ugr.es/pages/Data.
